# Prolonged confusional state as first manifestation of COVID‐19

**DOI:** 10.1002/acn3.51067

**Published:** 2020-07-06

**Authors:** Isabel Butt, Vijay Sawlani, Tarekegn Geberhiwot

**Affiliations:** ^1^ Southampton Medical school 12 University Road SO17 1BJ Southampton UK; ^2^ Department of Radiology Queen Elizabeth Hospital University of Birmingham Edgbaston Birmingham B15 2TH UK; ^3^ Institute of Metabolism and System Research Birmingham B15 2TH UK; ^4^ Department of Endocrinology University of Birmingham Queen Elizabeth Hospital Edgbaston Birmingham B15 2TH UK

## Abstract

A 77‐year‐old gentleman, normally fit and well, was admitted with acute confusion. On admission, Glasgow Coma Scale (GCS) was 14/15, vital signs were within the normal limits and bilateral crepitation at the lung base. Head CT scan was normal. CXR showed some air space opacification. Investigations revealed hyponatraemia, raised CRP, and positive for COVID‐19. Treated with antibiotics and intravenous saline, sodium returned to normal. Delirium remained unchanged 4 weeks post‐incidence. Neurological manifestations were documented in patients with COVID‐19; however no report has shown delirium as a primary manifestation. This case illustrates acute confusion may be the only presenting symptom of COVID‐19 without overt lung disease.

A 77‐year‐old Caucasian gentleman, who was normally fit and well, was admitted on March 29, 2020 with acute confusion after a fall on the stairs. Six days prior to his admission, he generally felt unwell, lethargic with unusual behavior wondering around at night. He has had no seizure, cough, temperature, or shortness of breath. He is known to have hypertension and was taking enalapril, indapamide, atorvastatin, aspirin, and lansoprazole.

On examination, the patient was awake but inattentive, disoriented to place, and time with GCS of 14/15. He was not in any form of cardiorespiratory distress and no evidence of injury to his head. The patient’s vital signs were all within the normal limits including oxygen saturation. His neurological examinations were unremarkable except confusion and examination of his chest revealed bilateral mid zone and basal crepitation. Computed tomography scan of his head was normal with no evidence of intra‐ or extra‐axial hemorrhage (Fig. 1). There was mild chronic small vessel ischemic changes. No cerebral atrophy and the basal ganglia, thalami, brainstem, CSF drainage pathway, and posterior fossa structures were within normal limits. The chest X‐ray showed air space opacification in the right lower zone and left peripheral mid and upper zone (Fig. 2). Biochemical investigations revealed low sodium at 123 mmol/l, raised C‐reactive protein at 44 mg/L, and plasma and urine osmolality were 252 and 291, respectively. The rest of his biochemical and hematological investigations including lactate and troponin were all within the normal limits. An oropharyngeal swab test using RT‐PCR was positive for severe acute respiratory syndrome coronavirus 2 (SARS‐CoV2/COVID‐19).

**Figure 1:**
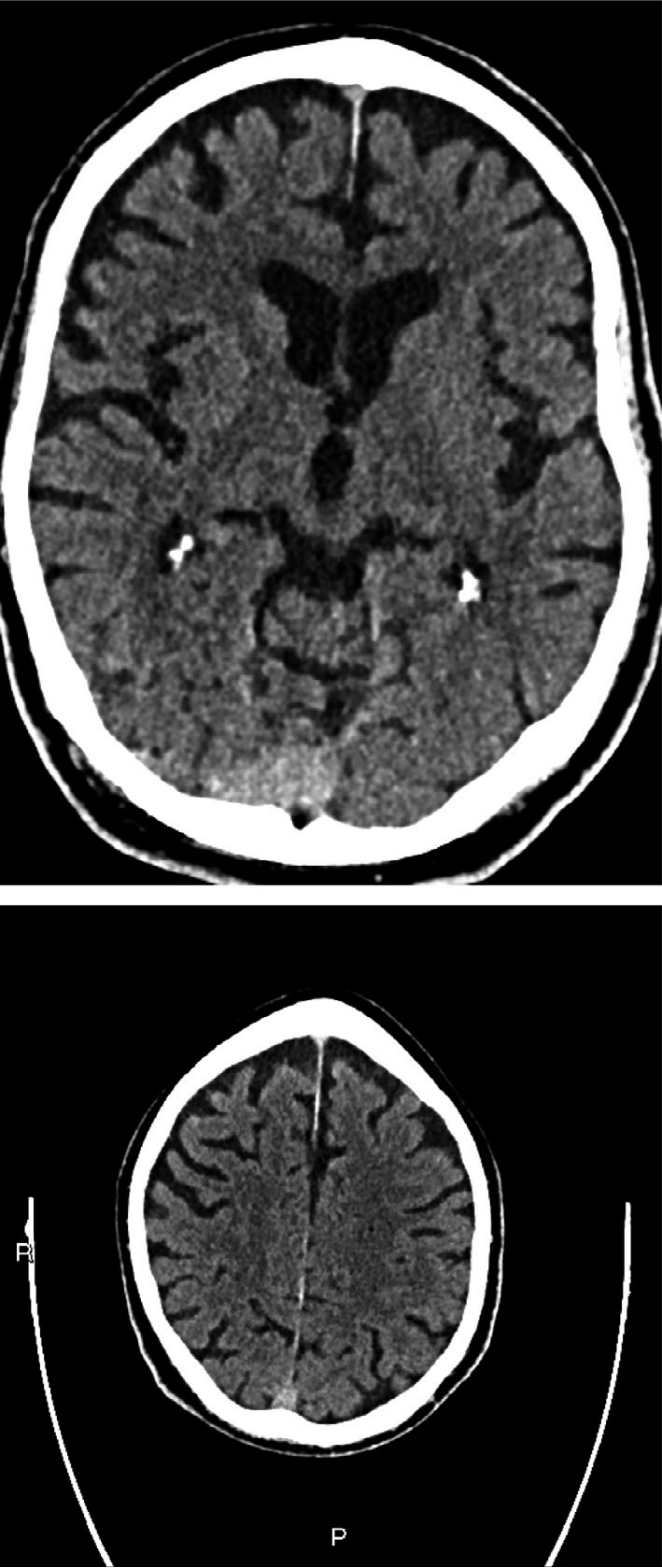
Plain CT head—there are mild chronic small vessel ischaemic changes seen. No cerebral atrophy. Basal ganglia, thalami, and brainstem structures are within normal limits. CSF drainage pathways are within normal limits. Brainstem and posterior fossa structures are within normal limits

**Figure 2:**
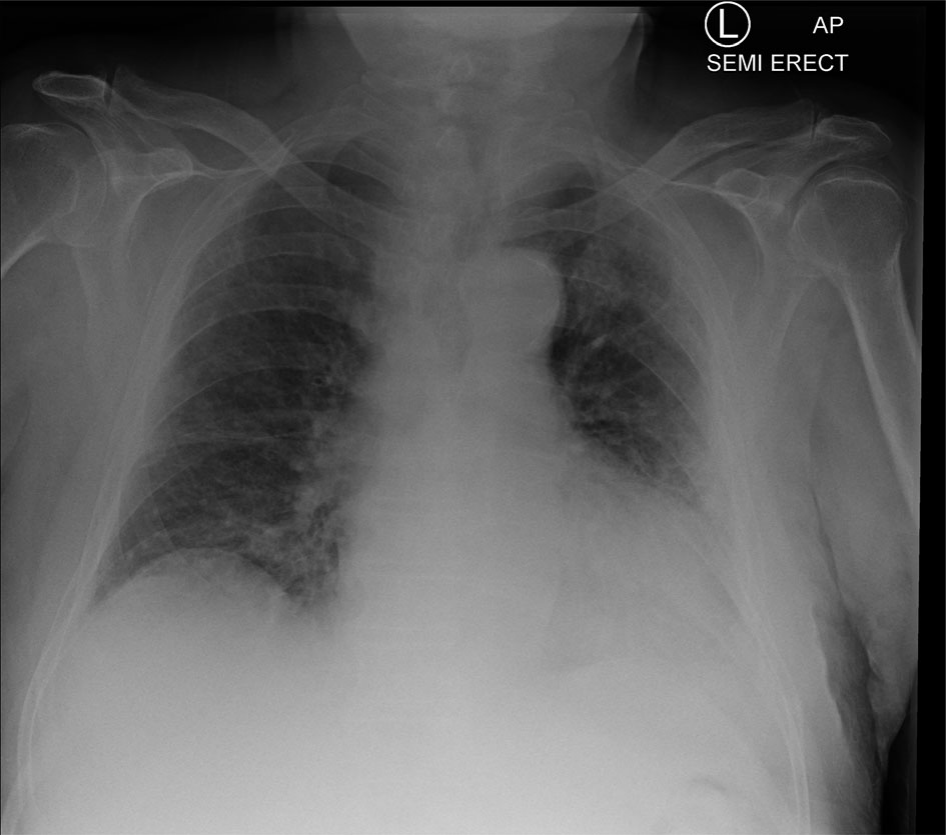
Chest X‐ray—air space opacification in the right lower zone and left peripheral mid and upper zone

Patient was treated with antibiotics, levofloxacin 500 mg for 5 days post‐admission, and intravenous 0.9% normal saline and his sodium over few days has gradually returned to normal. Up to 12% of patients with COVID‐19 were noted to have hyponatremia,^1^ and in this case, no other cause for low sodium or confusion was identified. An EEG during the course of his hospital stay was performed which showed no definite focal or generalized epileptiform activity. However, his prolonged confusion remained unchanged 4 weeks post the incidence and died on the April 18, 2020 due to hospital‐acquired infection. No postmortem examination was performed due to service pressures because of the pandemic. Our patient has been receiving angiotensin‐converting enzyme (ACE) inhibitor, enalapril. The interaction between the SARS‐CoV2 and ACE2 has been suggested as a potential virulence factor and there are concerns about the use of ACE inhibitors. The existing evidence is too limited to support or refute these concerns and yet we withheld his enalapril treatment.^2^

We think our patient has a primary central nervous system (CNS) disease related to COVID‐19 that led to delirium. Interestingly, he has never developed the respiratory symptoms of COVID‐19.

The cardinal feature of patients with COVID‐19 is respiratory symptoms.^3^ However, other organs involvement such as heart, gastrointestinal tract and nervous system has been documented.^4^ Mao et al report of 214 COVID‐19 patients, 36.4% were found to have neurologic manifestations at presentation, including headache, nausea, vomiting, confusion, ataxia, acute cerebrovascular disease, and seizures.^5^ In addition, a report of 99 COVID‐19 patients, 9% of patients presented with confusion on admission likely to be related to hypoxia or multiorgan damage instead of being a primary manifestation.^6^

We have described a case of COVID‐19 where acute confusion was the primary presentation without overt lung disease. Our case shed light that COVID‐19 infections may involve CNS in susceptible individuals and may contribute to overall morbidity and mortality. There is increasing evidence that coronavirus infections such as SARS‐CoV and now COVID‐19 may directly involve the CNS.^7^ The exact mechanism by which SARS‐CoV enters the CNS remains unknown, but may involve entry via the olfactory bulb with retrograde trans‐synaptic spread.^8^

As the global pandemic continues to unfold, COVID‐19 should be considered as a differential diagnosis in a patient with acute confusion without overt respiratory symptoms.

## Conflict of Interest

The authors declare that they have no conflict of interest.
